# Developing human rights based indicators to support country monitoring of rehabilitation services and programmes for people with disabilities: a study protocol

**DOI:** 10.1186/s12914-015-0063-x

**Published:** 2015-09-24

**Authors:** Dimitrios Skempes, Jerome Bickenbach

**Affiliations:** Department of Health Sciences and Health Policy, University of Lucerne and Swiss Paraplegic Research, Guido A. Zaech Institute (GZI), CH-6207 Nottwil, Switzerland; Human Rights in Patients Care Program, Association of Schools of Public Health in the European Region (ASPHER), Brussels, Belgium

**Keywords:** Health services for persons with disabilities, Rehabilitation, Human rights, Indicators, Accountability, Performance assessment

## Abstract

**Background:**

Rehabilitation care is fundamental to health and human dignity and a human right enshrined in the United Nations Convention on the Rights of Persons with Disabilities. The provision of rehabilitation is important for reducing the need for formal support and enabling persons with disabilities to lead an independent life. Increasingly scholars and advocacy groups voice concerns over the significant barriers facing people with disabilities in accessing appropriate and quality rehabilitation. A growing body of research highlights a “respond-need” gap in the provision of rehabilitation and assistive technologies and underscore the lack of indicators for assessing performance of rehabilitation systems and monitoring States compliance with human rights standards in rehabilitation service planning and programming. While research on human rights and health monitoring has increased exponentially over the last decade far too little attention has been paid to rehabilitation services. The proposed research aims to reduce this knowledge gap by developing a human rights based monitoring framework with indicators to support human rights accountability and performance assessment in rehabilitation.

**Methods/Design:**

Concept mapping, a stakeholder-driven approach will be used as the core method to identify rights based indicators and develop the rehabilitation services monitoring framework. Concept mapping requires participants from various stakeholders groups to generate a list of the potential indicators through on line brainstorming, sort the indicators for conceptual similarity into clusters and rate them against predefined criteria. Multidimensional scaling and hierarchical cluster data analysis will be performed to develop the monitoring framework while bridging analysis will provide useful insights about patterns of agreement or disagreement among participants views on indicators.

**Discussion:**

This study has the potential to influence future practices on data collection and measurement of compliance with human rights standards in rehabilitation service delivery and organization. The development of a valid and universally applicable set of indicators will have a profound impact on the design and implementation of evidence informed disability policies and programs as it can support countries in strengthening performance measurement through documentation of comparative information on rehabilitation care systems. Most importantly, the resulting indicators can be used by disabled people’s organizations as well as national and international institutions to define a minimal standard for monitoring and reporting progress on the implementation of the Convention on the Rights of Persons with Disabilities in the area of rehabilitation.

## Background

Disability is a major issue facing global health policy. It is estimated that one billion people around the world experience some significant degree of disability with 80 % of those living in low and middle income countries [1]. Figures from the Global Burden of Disease study indicate that Years lived with Disabilities (YLDs) increased from 537.6 million in 1990 to 764.8 million in 2013 with musculoskeletal, mental, and substance use disorders, neurological disorders, and chronic respiratory diseases being the main drivers of this increase [2]. Mortality rates of people with disabilities are also high. For example, people with spinal cord injury have an overall increased risk of mortality of 2.45 greater than that of the general population due to health problems associated with injury [3]. In addition to the health burden, the human and social impact of disability is diverse and far reaching. Recent research has found that disability is strongly correlated with poverty [4] depriving people with disabilities of their opportunity to access healthcare, education and other supports crucial for their well-being [5]. Moreover, people with disabilities often have their health rights violated and are far more likely to be denied healthcare or to be treated badly in the healthcare system. Consequently, health strategies aiming at promoting the human rights, independence and social inclusion of people with disabilities have become increasingly prominent in national and international policy agendas.

Rehabilitation – defined by the World Health Organization (WHO) as “a set of measures that assist individuals who experience or are likely to experience disability to achieve and maintain optimal functioning in interaction with their environment” [1] [p.96] – is a major strategy that addresses both the needs and rights of persons with impairments [6]. Although often neglected, rehabilitation is of paramount relevance to including people with disabilities in the community, and a valuable asset for health care systems. Acknowledging the importance of health-related rehabilitation in building human and social potential, as well as the significant barriers people with profound disability confront in attending individualized rehabilitation programmes, the United Nations Convention on the Rights of Persons with Disabilities (CRPD) [7] explicitly reaffirms the right to health for all people with disabilities (Article 25) and contains specific provisions that pertain to rehabilitation (Article 26), an integral but overlooked aspect of the right to health. The CRPD expressly stipulates the conditions and standards that should underpin the planning and delivery of rehabilitation services and programs in the area of health and specifies States’ legal obligations in guaranteeing equal and uninterrupted access to quality rehabilitation across the lifespan for all people with disabilities, including women, children and the aged [8].

Demographic and epidemiological trends suggest that the number of people who could benefit from interventions aiming to reduce dependency, optimize functions and promote social participation is set to increase due to, among other factors, population growth and ageing [2], thus putting significant pressure on health systems to strengthen and extend the provision of rehabilitative and other disability specific support services. The gap, however, between the need for rehabilitation treatment and its provision remains wide all over the world as shown in the World Report on Disability which documents systemic barriers to access to high quality and affordable rehabilitation [1]. These findings are consistent with more recent research that confirm the “respond-need” gap in the provision of rehabilitation across different contexts [9–13]. This mismatch can adversely affect the health and functional status of persons with disabilities, leading to catastrophic expenditures and poverty and exacerbating the social disadvantage associated with disability, such as stigma and discrimination, which in turn leads eventually to isolation and marginalization [14]. It is apparent that these inadequacies deprive people with disabling conditions from enjoying some of life’s fundamental experiences including their right to health and right to autonomy and participation. This needs to be addressed through coordinated, evidenced informed action at all levels. Effective monitoring of States’ compliance with the CRPD and comprehensive assessment of the performance of rehabilitation services is therefore imperative.

Health systems performance measurement and human rights monitoring require meaningful, objective and reliable indicators to quantify progress achieved. While it is true that the use of indicators depends critically on the availability and quality of data, in disability rights monitoring some indicators must be constructed *ex-novo* to drive the collection of data in consistency with the legal requirements set by the CRPD in areas where data are not available. Data collection on disability (and rehabilitation) is a key obligation of States stipulated in Article 31(1) of the CRPD, under which “*States Parties undertake to collect appropriate information, including statistical and research data, to enable them to formulate and implement policies to give effect to the present Convention*.” [7]. At present, the availability of accurate and reliable socioeconomic statistics on disability varies greatly across countries mainly as a result of the multitude of theoretical and methodological approaches for measuring disability and functioning in population based surveys [15–17]. The disagreement surrounding the definition of disability and inconsistencies in its operationalization yields a limited basis for comparative research and evaluation in this field [18] which makes monitoring the implementation of the CRPD practically difficult.

Driven by this absence of a unified framework for estimating disability prevalence and measuring environmental barriers to social participation, WHO is undertaking research to harmonize and advance data collection practices. This research, which is mostly guided by the WHO Disability Action Plan 2014–2021 “Better health for all people with disabilities” [19] aims at developing and testing a global population survey instrument for the collection of socio-epidemiological data on functioning [20]. While there is an overall consensus among health scientists that functioning is an important outcome for people with disabilities and functioning information is crucial for policy making [21, 22], international human rights law as expressed by the CRPD demands that States collect routine data on very specific aspects of health and rehabilitation services including data on barriers to access to rehabilitation. In particular, the CRPD emphasizes the tripartite duty of States to “organize, strengthen and extend comprehensive rehabilitation services and programs”, a duty that prompts attention to distinct dimensions of rehabilitation policy development and facets of service planning such as the availability, accessibility, affordability, acceptability and quality of rehabilitation services, products and technologies; the density of rehabilitation workforce; and the adequacy and readiness of rehabilitation facilities to deliver appropriate care. Additionally it is expected that signatories are in position to assess their progress in the implementation of the CRPD in the sphere of health governance as it pertains to rehabilitation. This requires concerted efforts to develop objective measures to monitor political and operational aspects of States’ participation in international cooperation mechanisms, transparency and corruption in the health system as well as the technical capacity of information systems for disability rights monitoring [23].

These aspects are sidelined in international initiatives to strengthen disability data and left out of States’ monitoring priorities. The narrowed approach to disability data collection that dictates current technocratic agendas [24] is inappropriate for guiding States in identifying rehabilitation needs and barriers [25] and falls short in serving the mandate of international health institutions to assist Member States in their efforts to fulfill their right to health responsibilities towards people with disabilities [26, 27].

Thus far, international efforts to strengthen data collection practices have limited their focus on survey based data and screening for disability through interview questions in population surveys ignoring other types of data from population based disease registries, hospital discharge records, and facility based reviews, all of which are essential for both evidence-based service planning and human rights reporting [28, 29]. This is because the predominant assumption about “better data” on disability is confined to health authorities being in position to report reliable incidence and prevalence estimates on disability in order to monitor equitable access to health and rehabilitation. But as Tobin notes “there is little practical benefit in gathering statistics on, for example the number of women or children who are denied access to healthcare if a state does not also collect information on the factors that are causing this denial and the cost of measures to ensure effective access” [30] [p.211]. Indeed, the discrepancy between definition and measurement of disability cannot and should not prevent researchers and health administrators from applying internationally accepted standards to collect comparable data on other important aspects of service planning such as the availability and density of rehabilitation workforce, an area where reliable information is severely lacking [31].

Global health leaders, agencies and independent expert groups have increasingly been calling upon States to intensify their efforts to collect appropriate data suitable to monitor the human rights status and situation of vulnerable groups and enhance their collaboration with specialized agencies to build the technical capacity required for health monitoring and human rights assessment. These calls have led WHO and the World Bank recently to propose a global framework for monitoring universal health coverage across the entire spectrum of essential health services [32]. The pilot testing of this framework, however revealed significant data gaps and problems in obtaining meaningful information to assess the coverage of discrete population groups for essential health services, especially for treatment and rehabilitation services [33]. The dearth of internationally acceptable and reliable measures to monitor rehabilitation services for people with disabilities is also evident in the reference list of core indicators published recently by WHO to facilitate health monitoring in the context of sustainable development goals [34]. In addition, and despite the fact that research on human rights monitoring and indicators in relation to health has increased exponentially over the last decade [35–37], including a relatively minor focus on the rights of people with disabilities [38–40], far too little attention has been paid to rehabilitation. As a result, the availability of indicators that could potentially be used to guide data collection for monitoring the implementation of the provisions of the CRPD pertinent to rehabilitation and contribute in rehabilitation services performance assessment is extremely narrow.

Currently we lack a validated international set of indicators, especially process indicators [41], that could assist in the examination of structural and organizational variations or failures in rehabilitation service planning and delivery, guide comparative rehabilitation systems analysis and ultimately facilitate evidence-based human rights reporting. Developing and agreeing on a robust set of metrics is the first step towards building strong rehabilitation services information systems at country level that can then prompt national and regional institutions to harmonize data collection practices in line with the requirements of the CRPD.

### Objectives and aims

Seeking to cover this knowledge gap, this paper proposes a study to develop a monitoring framework with indicators to support countries to assess progress in the implementation of the CRPD in the area of rehabilitation. Specifically the study aims to provide answers to the following research questions:

- Which indicators are appropriate for monitoring the implementation of the rehabilitation aspects of the CRPD?
- Which indicators are relatively more important and feasible and how the views of researchers, rehabilitation professionals and people with disabilities differ on the subject?
- What does a human rights monitoring framework for rehabilitation consists of, and how can it be put in practice?

The general approach to answering these questions and the methodological details of this study are explained in the Methods section. But before describing the research process, it is essential to define what a human rights based indicator is and understand some of the challenges associated with their development.

### Human rights indicators: conceptual and methodological challenges

In general terms indicators can be defined as numerical or textual values that provide information about a phenomenon. Indicators are essential elements of monitoring and evaluation systems that allow for assessment of progress towards the achievement of goals, enabling comparisons of different units or comparisons of the same unit at different time intervals. Unlike development and performance indicators, which follow programming principles, human rights indicators are explicitly anchored in human rights values and standards [42]. “Such indicators are meant to capture the extent to which the obligations flowing from those standards are being met and are yielding outcomes that can be associated with improved enjoyment of human rights” [43] [p.20]. Indicators that capture States compliance with human rights obligations are a necessary parallel to the health systems performance indicators allowing policy makers and administrators to make better choices to improve systems’ efficiency and responsiveness while respecting the law. The feedback provided by both performance and compliance indicators form the basis for consistent health systems accountability [44].

Methodologically, the development of human rights indicators is a challenging undertaking. In academic research, scholars have proposed and used many ways to accomplish the difficult task of indicators development, ranging from traditional research methods to more sophisticated approaches [45]. Currently, there is no universally accepted methodology for the development of indicators that would effectively satisfy both the principles of human rights monitoring and the scientific standards of validity and reliability [46]. Increasingly in legal and development practice, cross cutting indicators – indicators capturing both programmatic performance and legal compliance aspects – are elaborated in experts workshops were multiple stakeholders operationalize theoretical constructs and develop consensus on the key criteria for the selection of measurement tools to guide monitoring actions.

Regardless of the method being used to develop human rights indicators, one of the major challenges lies in the conceptualization of the treaty provisions (what to measure) and the operationalization of qualitative or textual information into measurable entities (how to measure). A thorough legal analysis is therefore essential to delineate the attributes of the right under examination and identify the key obligations of signatories to a specific treaty. These obligations must be then mapped onto indicators for which data are available and reliable. But as Fukuda Par notes, the criterion of measurability cannot dictate the selection of indicators since not all aspects relevant to human rights can be quantifiable or captured with measurement tools such as, people’s participation in the conduct of health affairs [47, 48]. Thus the main preoccupation is to identify those features or elements of the obligations of States that could be related to improved human rights enjoyment status and select proxy indicators that can capture not only quantitatively but also qualitatively the progressive efforts of duty bearers to comply with their obligations. In the end, the success and legitimacy of human rights indicators will depend on (i) whether the process of their development has been truly participatory and based on human rights principles; (ii) whether the method used is scientifically valid and reliable; and (iii) their usefulness as tools of accountability [30]. As yet, theoretical approaches and frameworks that have been proposed to guide the process of developing rights based indicators [43, 49, 50] fail to describe a rigorous analytic approach for their elaboration that would satisfy the above criteria. Such frameworks are nonetheless useful conceptual contributions that have advanced our understanding of the purpose of human rights monitoring and the inherent limitations in the selection of appropriate indicators that can be used as input to a more structured process of devising such measures.

## Methods/Design

For developing the rehabilitation monitoring framework and identifying appropriate human rights-based indicators we propose to carry out a Group Concept Mapping study (GCM) that is underpinned by the methodological principles of structured conceptualization [51]. GCM is a mixed methods participatory approach that combines group processes such as brainstorming; sorting and rating; and group interpretation, with a sequence of multivariate statistical analyses. GCM “facilitates the collection of input from a broad and diverse array of stakeholder groups and/or other data sources, in virtually any setting in which a group issue or need requires definition, planning and evaluation, and it enables feedback on these data to participants” [52] [p.1]. Concept mapping techniques have been used widely in public health research [53, 54], including in projects aiming to identify the key conceptual domains that define a monitoring and evaluation framework [55], to develop potential outcome measures and explore the structural relationships among these domains or measures [56–59]. As opposed to other research methods such as focus groups and consensus meetings, GCM represents a systematic process that combines the strengths of qualitative research with the analytic rigor of multivariate statistical methods to produce results that are both valid and reliable [60]. It is therefore believed that such an approach could be highly valuable in the ex-novo development of cross-cutting indicators for rehabilitation as it is also pragmatic and encourages participant engagement across all stages of the research process.

### Context and setting

Conceptually, the study will be guided by key international legal instruments pertaining to disability and rehabilitation, specifically the CRPD and particularly Article 25 (Health), Article 26 (Rehabilitation) and Article 31 (Data collection and Statistics). An analysis of the obligations of States under the CRPD with respect to rehabilitation and its resulting framework will serve as a conceptual platform for identifying potential indicators in key human rights commitment areas [8]. This platform will be further strengthened and informed by key guiding documents on the development of indicators for human rights monitoring and reporting [50, 61] and health system performance measurement [62, 63]. This information will be collated in a draft document along with a compendium of potential measures drawn from the scientific literature which will serve as background to a highly structure, global experts survey conducted online through the World Wide Web.

### Participants sampling and recruitment

There is no limit on the number of people who can participate in GCM. Based on a pooled analysis of 69 concept mapping studies Kane et al. found a range of 20–649 participants with an average of 75 participants completing the rating phase [60]. Assuming a highly conservative response rate of 30 % in the brainstorming phase and modest attrition rates of 40 % thereafter we aim to recruit *n* = 150 participants through purposive and self-selection from the following stakeholder groups: (i) *n* = 50 rehabilitation professionals and service managers, (ii) *n* = 50 researchers with expertise in various disciplines such as global health law, health services research and rehabilitation medicine, and (iii) *n* = 50 people with disabilities and representatives of disabled people’s organizations. This will result in *n* = 27 participants sorting and rating the indicators which is above the minimum number recommended for ensuring the validity and reliability of the study results (*n* = 15) [64].

For recruiting experts in the study the investigators will contact international professional organizations and societies, disabled people’s organizations and individual academics and peers asking them to participate in the study and nominate additional experts for participation. A pool of participants who fulfil the criteria for participation (>4 years of professional experience) will then be stratified by stakeholder group. A random sample will be drawn from the experts pool for each stakeholder group and sent an email invitation to participate in the survey. Invitees who decline the invitation are replaced by other randomly drawn experts from the same expert pool.

### Data collection and data analysis

In GCM data can be collected either in paper form during pre-scheduled workshops or remotely through dedicated web based platforms. In this study data collection will be facilitated by the Concept Systems Global MAX™, a web based software specifically designed to facilitate concept mapping activities [65]. Data pertaining to employment status and occupation will be collected by all individuals who will agree to participate in the study. Participants will be asked to provide information regarding their occupation, the type of organization they are affiliated with, their field of expertise, number of years of professional experience and their geographic location. This information will make it possible to compare the views of respondents who belong to different stakeholder groups. For example we will explore how the perceptions of rehabilitation professionals and providers differ from the views of those representing disabled people’s organizations about indicators or how the views of researchers differ from the views of disabled people’s representatives.

**Fig. 1:**
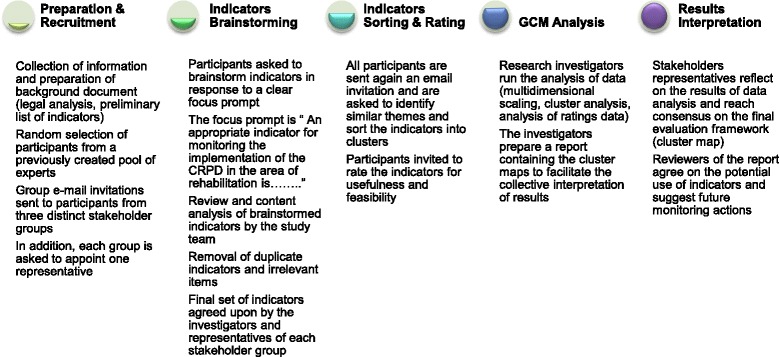
Analytic process for the development and selection of human rights indicators. Detailed legend: The participatory research process for developing and selecting rights based rehabilitation indicators involves five sequential steps: experts consultation preparation including sampling and recruitment of stakeholders, indicators brainstorming, indicators sorting and rating, participant data analysis, collective interpretation of results. Abbreviations: CRPD, Convention on the Rights of Persons with Disabilities; GCM, Group Concept Mapping

Based on the principles of GCM [52] a sequential process will be followed for the collection and analysis of participant data. The stages of this process are presented in Fig. 1 and the activities following the preparations outlined above are described in more detail in the text below:

#### Indicators brainstorming

At this stage, a focus prompt – an incomplete sentence designed to invite people to complete it – will be used to elicit participants’ best ideas and expertise on the topic. The focus prompt is: *“An appropriate indicator for monitoring the implementation of the CRPD in the area of rehabilitation is…”* Using this question, participants will brainstorm a wide array of measures which will result in a long unfiltered list of potential indicators. After the conclusion of the brainstorming session, the research investigators will review and content analyze the brainstormed indicators to remove duplicate or other irrelevant statements and agree with stakeholder representatives on a final set of measures for further elaboration.

#### Indicators sorting and rating

In the first data collection activity of this phase all participants will be invited by email to organize the indicators into categories in a way that makes sense to them and thus identify the conceptual building blocks for the rehabilitation monitoring framework. Participants will also rate the indicators for importance and feasibility, thus allowing patterns of agreement and/disagreement among different stakeholder groups to be identified.

#### Concept mapping data analysis

Data analysis will be performed using special functions of the Concept Systems Global MAX™ software [65]. Multidimensional scaling will be used to examine similarities of responses in participant data and hierarchical cluster analysis to set boundaries around indicators that share a strong degree of similarity. The result is a series of easily readable cluster maps. These maps will show the relationship between indicators, and the clustering of similar indicators into categories. Bridging analysis of the indicators ratings data will also allow the investigation of patterns of agreement and/disagreement between stakeholder groups. Finally the values assigned by participants to each indicator will be analyzed to illustrate a concrete set of commonly agreed actionable indicators for monitoring the implementation of the CRPD in rehabilitation.

#### Results interpretation

These analyses will finally lead to a general discussion of the reasonability of the resulting monitoring framework and its implications for human rights compliance and rehabilitation systems performance assessment. Specifically, in the final phase of the GCM, stakeholder groups' representatives and experts in public health monitoring will be invited to review the clusters of indicators and the underlying relationships between them and to collectively interpret the results in a way that can drive evidence based action.

### Ethics clearance

Ethics review and clearance has been obtained for this project by the Ethics Commission for Northwest and Central Switzerland.

## Discussion

This study aims to reduce the information gap in the assessment of performance of rehabilitation services and programmes for people with disabilities. It also has the potential to influence future practices on data collection and measurement of compliance with human rights standards in the delivery and organization of rehabilitation care. The development of a valid and universally applicable set of indicators will have a profound impact on decision making and implementation of evidence informed policies for rehabilitation at national and global level. Furthermore, the resulting monitoring framework can support the documentation of comparative information on national rehabilitation care systems and could be used as a minimal standard for monitoring and reporting progress on the implementation of the CRPD in the area of rehabilitation.

### Strengths and limitations

A unique feature and major strength of the GCM is that it employs a truly participatory process for developing the indicators. Unlike other research methods the approach to data collection and elaboration is highly structured and under the control of the participants. Specifically and significantly, GCM allows stakeholders to contribute their expert knowledge by identify relevant metrics, participate in the interpretation of their perceptions and elaborate on their potential use of the resulting evaluation framework. An additional strength lies in the use of advanced statistical techniques to provide useful insights about the relationships of the ideas of different stakeholder groups, which provides rigor and enhances the credibility of information obtained during qualitative research processes. This results in visual representations of the indicators in the form of maps that are relatively easy to understand and interpret in the subsequent phases of the research process.

Alternative approaches that have been used for the development or selection of indicators in health research either lack scientific credibility [66] or adopt a narrowed definition of citizens’ participation in research, one that considers them merely as data providers and leaves very little room for them to integrate their perspectives in the interpretation of study results. But according to WHO, the participatory development of indicators in health systems performance assessment is a matter of good and inclusive governance and “increases the sense of ownership of the results as well as the chances of seeing policy action based on these results” [62] [p.17]. Moreover, the genuine participation of persons with disabilities in the conduct of health affairs is both an operative principle and an obligation under the CRPD. As such, a rights based approach to the development of rehabilitation indicators demands that persons with disabilities participate as collaborators in research processes, including not only in the formulation of research questions and data generation but also data interpretation [67, 68]. The participation of those affected by the outcome of the evaluations in the development of indicators may be costly and time consuming [69], however is the only way to balance the interests of multiple actors in accountability monitoring and performance measurement and ensure rigor and credibility of monitoring frameworks in the eye of all stakeholders involved [70].

As with conducting on line surveys, GCM presents a number of challenges for the investigators to address when collecting, organizing and interpreting the data. Remote data collection in concept mapping may allow researchers to access large and geographically distributed samples of participants, but they carry with them specific limitations when compared to the traditional paper based collection of data. These include the risk of a low response rate and a resource intensive process for collecting and analyzing the data [52]. In order to eliminate potential threats to validity of the results, the study team intends to apply evidence based strategies aiming to strengthen participants’ engagement in GCM by increasing, for example, the frequency of email reminders and personalizing the communication between respondents and researchers [71, 72].

### Implications

The results of this study are relevant to key stakeholders at both the national and international level in a variety of ways. First, the resulting indicators set will equip national public health and statistical authorities and human rights institutions with a valuable tool to monitor States’ compliance with international human rights law standards in the organization and delivery of rehabilitation services. In so doing, competent authorities may choose to either adjust their own data collection and monitoring systems to include human rights based indicators or use the new metrics separately on an ad hoc basis during periodic country performance reviews. Second, the results will be relevant to researchers, policy analysts, advocates and global health stakeholders, providing them with an evaluation framework and menu of newly created, evidence based measures that can be used to standardize the assessment of rehabilitation services and programs and facilitate comparative evaluation research.

In addition to satisfying the need for human rights monitoring and performance assessment, the rehabilitation indicators will be extremely valuable for UN agencies, Member States and other international stakeholders. The metrics proposed by this study will augment existing quantitative and qualitative measures thus offering a more comprehensive evaluation toolbox that WHO and other organizations can use in their programmatic activities, as for example to assess progress in the implementation of the WHO Global Disability Action Plan. Finally, the indicators set will be based on the legal requirements of the CRPD that represents a nearly universal consensus as it has been ratified by nearly 160 countries in the world. This makes the set of indicators applicable in all contexts and thus might be an appropriate start for the development of specific human rights monitoring guidelines for Article 25 and 26 by the UN committee responsible for the oversight of the CRPD.

Lastly, from a consumer’s perspective, the long standing misapprehensions surrounding the goals of rehabilitation and the contested role of health professionals in disability management make the results of this research even more important. Since people with disabilities have been traditionally excluded from decision making in rehabilitation care planning and programming, rights based indicators will prompt rehabilitation professionals and service designers to reconsider oppressing attitudes and approaches in their everyday clinical practice and pay more attention to human rights issues in patients care [73]. More importantly, the indicators will provide disabled people’s organization with a powerful tool to hold governments accountable for the implementation of key provisions of the CRPD as regards to national rehabilitation policy. This will empower a historically vulnerable group of our society to advocate for their own health and rights in a context of increased competition for resources and political attention.

## Conclusion

The results of this study will provide the global health and disability community with a preliminary set of cross-cutting indicators for assessing performance of and monitoring compliance with human rights standards in rehabilitation service planning and delivery. It will help identify gaps in quantitative data and other qualitative information that are essential for the comparative evaluation of national rehabilitation care systems. This will stimulate action toward the expansion of information gathering on rehabilitation services and further research on the development, testing and validation of indicators for various tracer conditions. The study described in this paper is a major step toward advancing the current practice of rehabilitation systems performance comparison and promoting evidence-based human rights monitoring and reporting.

## Abbreviations

CRPD: Convention on the Rights of Persons with Disabilities
GCM: Group Concept Mapping
UN: United Nations
WHO: World Health Organization
YLDs: Years Lived with Disability
